# Supplementary material for: Nationwide introduction of HPV vaccine in Zimbabwe 2018–2019: Experiences with multiple cohort vaccination delivery
Source: PLOS Glob Public Health. 2022 Apr 6;2(4):e0000101. doi: 10.1371/journal.pgph.0000101 (PMC10021852; doi:10.1371/journal.pgph.0000101)
Supplement: S1 File — (PDF) [file pgph.0000101.s001.pdf]

Zimbabwe: Documentation of national introduction interview guide – National level

The following designations indicate the organization of primary target interviewee:

- EPI: Ministry of Health Immunization Program Manager
- MOF: Ministry of Finance Immunization Focal Point
- WHO: World Health Organization Immunization Focal Point
- UNICEF: UNICEF Immunization Focal Point
- MoE: Ministry of Education HPV Focal Point

Oral consent statement:

Do you agree to information collected in this interview being used for documentation of the HPV vaccine introduction in Zimbabwe, consolidated lessons learned across several countries and/or a manuscript for sharing of lessons learned? No identifying information will be collected and interviewees/organizations will not be identified by their responses.

☐ Yes
☐ No

DECISION-MAKING

Drivers for Decision to introduce HPV vaccine into national immunization program

EPI	1. What evidence was used in the decision to introduce HPV vaccine? (prompts: national estimate of burden of cervical cancer, social or economic costs of cervical cancer, cost effectiveness studies, perception of risk/acceptability among public/health workers/medical community, access to health care among girls/vulnerable groups, vaccine effectiveness studies)
EPI	2. How did global supply availability influence the decision to introduce nationally and vaccine choice? (choice between Cervarix and Gardasil)
EPI	3. Was HPV vaccine introduction considered a political priority? Why?
EPI	4. How did experience during the demo influence the decision to scale up?
EPI	5. What is the current availability and use of other cervical cancer prevention methods (prompts: screening and treatment)? Plans for future scale up?
EPI	6. Were alternative interventions considered (cervical cancer screening and treatment, adolescent health interventions) when making the decision to introduce HPV vaccine?
EPI	7. Describe the status and influence of a National Cancer (or Cervical Cancer) Strategic Plan in HPV introduction?
EPI	8. Who were the main stakeholders in the decision-making process and what were their roles? Which had the strongest influence?
EPI	9. Does the country have a NITAG? How often does it meet? Who are the members?

EPI	10. How long did the decision-making process take? (time discussions began to time application for vaccine introduction was submitted)
EPI	11. What guidance, tools, templates from the global level were used during decision-making? (What additional guidance, tools, templates would be helpful?)
ALL	12. What are the best practices/challenges in decision-making? Any advice for other countries currently in this process?

Programmatic

EPI	13. How were programmatic decisions made? (Was a stakeholder meeting held?)
EPI	1. Confirm decisions made for each of the programmatic considerations identified through desk review.<br>a. Vaccine choice- Cervarix or Gardasil<br>b. Estimating target population (what data source)<br>c. Choosing cohorts<br>d. Dosing schedule<br>e. Defining eligibility and tracking systems<br>f. Primary delivery strategy<br>g. Mop up activities?<br>h. Strategies for reaching out of school girls and special populations (HIV+)<br>2. Describe why the above considerations were chosen
EPI	3. Are there any concerns with decisions made? (prompts: annual dosing, tracking systems, target population estimates)
EPI	4. Which considerations to the immunization programme were most important when deciding to introduce HPV vaccine? (prompts: feasibility, logistics, workforce capacity, cold chain, supply availability)<br>a. How did global supply availability impact the decision to introduce nationally and vaccine choice?
EPI	5. Was the issue of consent important when making the decision to introduce HPV vaccine?

Financing

EPI<br>MOF	14. What funding sources were considered during decision-making?
EPI<br>MOF	15. Who was involved in financial discussions (i.e., which sectors within the government was involved)?
EPI<br>MOF	16. What percentage of vaccination delivery costs for HPV vaccine introduction were covered by the government? (other sources?)
EPI<br>MOF	17. Was a funding gap for HPV vaccine introduction anticipated (give approximate percentage)? If so, how was this addressed?
EPI<br>MOF	18. Was financial sustainability discussed during decision-making (i.e., how does the government intend to fund HPV vaccination on an annual basis)?
EPI<br>MOF	19. How effective was financing for HPV introduction during dose 1? (Prompts: Was funding received sufficient for activities planned? Were funds received on time? How were they distributed by level of the health system for use?)

Budgeting

EPI<br>MOF	20. Did information from the demonstration project costing study inform the budget development process for the HPV vaccine national introduction?
EPI<br>MOF	21. Who was involved with budgeting development process for the HPV vaccine national introduction (i.e., which government sectors and non-governmental partners)?
EPI<br>MOF	22. What other financial data was used to derive a budget plan for the HPV vaccine national introduction?
EPI<br>MOF	23. What was the budget allocation (%) by level of the health system (e.g., % allocated by national, province, district, health facility)?
EPI<br>MOF	24. Were any budget adjustments made after dose 1 leading into preparation for dose 2?

Other

EPI<br>WHO	25. What are the best practices/challenges in decision-making? Any advice for other countries currently in this process?
EPI<br>WHO	26. Anything else about decision-making that you would like to share?

PLANNING & IMPLEMENTATION

Coordination and application

EPI<br>WHO	27. What was the process for completion of the Gavi application? (Prompts: Was a consultant hired? What was the approval process? How long did the process take?
EPI<br>WHO	28. Describe any obstacles or setbacks in the HPV vaccine application process.

Vaccine licensure and importation

EPI<br>UNICEF	29. Describe the licensing process through the NRA or similar body.
EPI<br>UNICEF	30. Were there any barriers to importation of vaccine into the country?

Planning

EPI	31. Describe microplanning process in Zimbabwe? (Prompts: Was a microplanning meeting convened and who attended? How were target populations/vaccine requirements/human resource needs identified? Were schools and health facilities mapped? What tools/guides were used?)
EPI	32. How was the target population determined? (Prompts: top-down vs bottom up?)
EPI<br>UNICEF	33. Were changes to the cold chain system required before introduction of HPV vaccine? How were cold chain requirements calculated?

EPI	34. How was waste management done? Were changes to the waste management system required before introduction of HPV vaccine?
EPI	35. What were the findings of the pre-introduction readiness assessment?<br>a. Was the readiness assessment informative to the program<br>b. What changes were made prior to introduction?<br>c. Would you recommend a readiness assessment to other countries planning for national HPV vaccine introduction?

Training

EPI	36. Describe the training process for health care workers (Prompts: How many days was the training? What topics were covered in training? How many staff in each training? (trainer to trainee ratio) Was cascade training used? Was HPV training combined with another training? Were training materials created in country or were global templates adapted?)
EPI<br>MOE	37. Describe the training process for school personnel.
EPI	38. Any other trainings? (community leaders or religious leaders?)

Communication

EPI<br>UNICEF	39. Describe communication/social mobilization activities? (Prompts: Was there a launch? Who attended? Who delivered key messages? When did communication activities begin? Was there a written communication plan?)
EPI<br>UNICEF	40. How were communication strategies decided? Was formative research done to inform communication strategies?
EPI<br>UNICEF	41. Was a risk/crisis communication plan developed? Where there any communication crises/ rumors/events that occurred? How were issues overcome?
EPI<br>UNICEF	42. How did parents give permission to vaccinate? (opt-in or opt-out) How were parents informed about vaccination activities or accessed for consent?

Implementation

EPI	43. How many staff used to deliver the vaccine? Did this differ by vaccination site? Were additional staff used for HPV vaccination or were they the same staff that delivery EPI vaccines? How are staff supervised during HPV vaccine implementation?
EPI	44. What was staff workload like during HPV vaccination? How long did vaccination activities take? Did this affect normal health worker activities?
EPI<br>MOE	45. If school delivery was used, what was the role of teachers and educational staff in delivery?
EPI	46. How was vaccine supplied to delivery sites? Have there been any stock outs?
ALL	47. What are the differences between dose 1 and dose 2, in your opinion?

Monitoring

EPI	48. Describe AEFI monitoring (Prompts: Was a new AEFI monitoring system created for HPV vaccine or were existing procedures used? Describe the process for post-vaccination observation.)
EPI	49. Describe the immunization tracking. (Prompts: Where immunization forms, health records, and registers updated or created new? How were girls tracked for 2nd dose vaccination? How were missed girls identified?) (make sure this section is detailed and accurately diagrammed as it will be used in data quality assessment)
EPI<br>WHO	50. How is coverage monitored? How often? Which sources are used for the denominator? Why was this source selected?

Other

EPI	51. What are the best practices/challenges in planning and implementation?
EPI	52. What guidance, tools, templates from the global level were used during planning and implementation? (What additional guidance, tools, templates would be helpful?)

Integration & Sustainability

EPI<br><br>MOE	53. How has HPV vaccination been integrated with other public health interventions for adolescents? (Prompts: training, delivery, communication, etc.) Has a school health platform been established? Describe any plans for integrating HPV vaccination with other programs in the future.
EPI<br>MOF	54. What are plans for financing the HPV program in the future?
EPI	55. What are the differences between the demo and national introduction so far?

Archive of country-developed tools and materials

EPI	56. Are you willing to share tools and materials developed used during HPV introduction to be archived and disseminated through global channels?	☐Yes<br><br>☐ No
	Category:<br><br>☐ Field guides<br><br>☐ Operational guidelines<br><br>☐ Decision making meeting minutes (ICC, NITAG)<br><br>☐ Costing tools<br><br>☐ Budgeting tools	List and location of materials (check if copy is received by study team):

	☐ Training materials<br>☐ Communication Plan<br>☐ Job aids<br>☐ Vaccination cards<br>☐ Registers<br>☐ Tally sheets<br>☐ Brochures<br>☐ Posters<br>☐ Leaflets<br>☐ Press releases<br>☐ ________________________________	

Appendix 2.5B HPV Post-Introduction Evaluation Questionnaire — Health worker at vaccination session

Date of interview: ______________________________ Name of interviewer: ________________________

This questionnaire was conducted at:

Province: ____

District: ____

Name of School : ____

Name(s) and title(s) of person(s) interviewed (please list all persons that you interviewed):

Persons responsible for vaccinations (or their deputy) should be interviewed

Name: ____ *Title:* ______________________________________

Cell No: ___________________________________

Name: ____ *Title:* ______________________________________

Cell No: ___________________________________

Name: ____ *Title:* ______________________________________

Cell No: ___________________________________

Contact details of most senior person:

Telephone: ____________________________________ *E-mail address:* _____________________________

★ Denotes suggested Key Findings.

	**1. TRAINING**	
GEN	1. Describe the training you received?<br>(ask if they received refresher training before dose 2)	
GEN	2. What specific training did you receive on the administration of the HPV vaccine?	**Check all mentioned**<br>☐ Correct administration (intramuscular, shake well before use)<br>☐ Correct technique (intramuscular injection in deltoid region of the upper arm or in the higher anterolateral area of the thigh)<br>☐ Introduction to HPV infection and cervical cancer<br>☐ HPV vaccine attributes and storage conditions<br>☐ HPV vaccine eligibility and contraindications<br>☐ HPV vaccine administration<br>☐ Recording and monitoring of HPV vaccine doses<br>☐ Social mobilization about HPV with key stakeholders<br>☐ Dealing with care of adolescent patients<br>☐ Identification and reporting of AEFIs<br>☐ Other, specify:_____________________________<br>☐ Don't know
GEN	3. Do you think there are any ways in which the training could be improved for next time?	☐ Yes ☐ No ☐ Don't know<br>If yes, please describe____________________________
GEN	4. Overall, were you satisfied with the training provided?	☐ Yes ☐ No ☐ Don't know<br>Explain:____<br>★ **Key Finding: Satisfaction with training?**
	**2. VACCINE COVERAGE**	
GEN	5. Is HPV vaccine recorded in a personal vaccination card?	☐ Yes ☐ No ☐ Don't know
GEN	6. What is the target group for HPV vaccination?<br>*Answer:*<br>*First cohort (2018): 10-14 in and out of school*<br>*Second cohort (2019): Grade 5 in school/age 10 out of school*	First Cohort, receiving dose 2 (2018):<br>Target age group:____________________<br>Target out of school ___________________<br>Second Cohort, receiving dose 1 (2019):<br>Target grade/class:_____________________<br>Target out of school ___________________
GEN	7. What is the size of target population for HPV vaccination (in level being interviewed) during this campaign?<br>What is the source of these figures?	First Cohort, receiving dose 2 (2018):<br>Number of girls age 10-14 in 2018:___________________<br>Second Cohort, receiving dose 1 (2019):<br>Number of girls targeted in grade 5:____________________<br>Target out of school age 10: __________________

GEN	8. How often do you report HPV immunization data to the district?<br>**Ask to see a report.**	☐ Daily during campaign<br>☐ After all doses have been administered<br>☐ After mop up<br>☐ Don't know<br>☐ Other, Specify______________________________
GEN	9. Are immunization reporting and recording tools and forms for HPV vaccine available?	☐ Yes, all forms developed<br>☐ Yes, most tools, except (please list)____________<br>☐ No<br>☐ Don't know
**3. VACCINE DELIVERY**		
GEN	10. Are there any problems implementing the HPV delivery strategies?<br><br>Note: For example – out of school girls not being reached; schools/heads refusing vaccination teams; absenteeism on day of vaccination.	☐ Yes ☐ No<br>If yes, please describe both problems and solutions:
GEN	11. What methods were used to ensure girls return for their subsequent dose(s)?	☐ Note date to return on vaccination card<br>☐ Inform girls when to come for second dose<br>☐ Other, specify______________________
	12. Did you have people outside target group wanting to be vaccinated?<br><br>*Note:*<br>*For in school: outside the grade 5 or 10-14 years ;*<br>*For out of school older or younger than 10-14*	☐ Yes ☐ No<br><br>What did you do and say to them?<br>________________________________
**4. COLD-CHAIN MANAGEMENT**		
GEN	13. Were any problems with the cold chain identified after the introduction of the HPV vaccine? If yes, what were the problems and how have the problems been addressed?	☐ No problems<br>☐ Use of ice packs<br>☐ Inadequate space<br>☐ Frozen vaccine<br>☐ Malfunctioning refrigerators<br>☐ Power supply/fuel shortage<br>☐ Other (specify)<br>☐ How resolved? ____________________________<br><br>★ **Key Finding: Percentage health facilities observed or reported problems with the cold chain**
**5. VACCINE MANAGEMENT, TRANSPORT & LOGISTICS**		
GEN	14. Did you run out HPV vaccine, or vaccine supplies in the past six months?	☐ Yes<br>☐ No<br>Describe:<br><br>★ **Key Finding: Percentage of health facilities reporting vaccine or supply stock out in last six months**

GEN	15. Did you receive your vaccine quantities aligned with injection supplies when distributed (i.e. bundling)?	☐ Yes ☐ No
GEN	16. When should an open vial of Cervarix be discarded?<br><br>*Open cervarix vials (unused doses) should be discarded at the end of the vaccination session or after 6 hours (whichever comes first)*	☐ At the end of the vaccination session<br>☐ 6 hours after opening<br>☐ Other:_________________________
**6. WASTE MANAGEMENT AND INJECTION SAFETY**		
GEN	17. How does your team manage waste during the campaign?	Describe:
GEN	18. Have you experienced any problems with your waste-management system?<br><br>**Observe waste disposal site. Record findings in Annex 2.6 Section 3.**	☐ Yes ☐ No<br>If yes, explain__________________________
**7. VACCINE WASTAGE**		
GEN	19. What formula is used to calculate vaccine wastage and what is the source of the data?<br><br>**Ask for wastage report.**<br><br>**Vaccine Wastage Formula:**<br>$\frac{\textit{Doses Consumed - Doses administered}}{\textit{Doses Consumed}}$<br><br>Include the source of the numerator (doses administered) and denominator (target population).	☐ Vaccine wastage not calculated<br>Formula:<br>Data source, numerator__________________<br>Data source, denominator_________________<br><br>Is formula provided correct?<br>☐ Yes ☐ No<br><br>Source of data:<br>☐ summary sheets<br>☐ Other<br><br>★ **Key Finding: Wastage report on site? ☐ Yes ☐ No**
**8. ADVERSE EVENTS FOLLOWING IMMUNIZATION (AEFI)**		
GEN	20. Have you reported any AEFIs for the HPV vaccine during this campaign?	☐ Yes ☐ No ☐ Don't know<br><br>If yes:<br><br>Describe: ____________________________
**9. ADVOCACY, COMMUNICATION & ACCEPTANCE**		
GEN	21. Did you experience any resistance from the community regarding the HPV vaccine?	☐ Yes ☐ No ☐ Don't know<br>If yes, from who in particular? ____________________<br>How many girls were in this group of people who expressed resistance?______________<br>How many girls were finally vaccinated?__________<br><br>★ **Key Finding: Percentage of Health Facilities indicating resistance to the HPV vaccine?**

GEN	22. Were there rumours affecting HPV vaccination?	☐ Yes ☐ No ☐ Don't know<br>If yes, describe the rumours? ____________________________<br>____
**10. HEALTH-CARE WORKER KNOWLEDGE**		
GEN	23. What is the immunization schedule for the HPV vaccine?<br>*In training: 6-15 months or 1 year*	Schedule:<br>Dose 1 ____________<br>Dose 2 ____________
GEN	24. Please explain the correct way to administer the HPV vaccine.	**Check all mentioned**<br>☐ Correct administration (intramuscular, shake well before use)<br>☐ Correct technique (intramuscular injection in deltoid region of the upper arm or in the higher anterolateral area of the thigh)<br>☐ Other, specify__________________________________<br>☐ Don't know
GEN	25. Have you or other staff experienced any problems with administering HPV vaccine?	Record any problems mentioned
GEN	26. What disease(s) does the HPV vaccine being used in Zimbabwe (Cervarix) prevent?<br>**Interviewer: Do not mention these diseases to the interviewee.**<br>*HPV vaccine prevents cervical, vulvar, vaginal and anal cancer (caused by HPV types 16 and 18);*	☐ Cervical cancer<br>☐ Other cancers, specify:_____________________________<br>☐ Don't know<br>★ **Key Finding: Percentage HCWs who knew what diseases the HPV vaccine prevents?**
GEN	27. What information do you provide to girls (and their parents if accompanied) before and after vaccination with the HPV vaccine?	**Check if mentioned — don't prompt but can tell afterwards**<br>☐ Name of the vaccine<br>☐ Diseases it protects against<br>☐ Benefits to the girl<br>☐ Vaccine schedule/when to return<br>☐ Normal side effects<br>☐ What side effects they should return for<br>☐ Bring vaccination card to next visit<br>☐ Other health messages – related to cervical cancer prevention (specify)<br>Two or more mentioned? ☐ Yes ☐ No<br>★ **Key Finding: Percentage HCWs providing two or more accurate pieces of information to girls and/or parents?**

11. GENERAL IMPRESSIONS		
GEN	28. In your opinion, was the introduction of the HPV vaccine a smooth process or problematic? Please explain.	Please check one that best describes the implementation:<br>☐ Very smooth. No problems<br>☐ Generally smooth, minor problems.<br>Please explain ___<br>☐ Somewhat smooth, some major problems.<br>Please explain ___<br>☐ Not smooth at all. Major problems.<br>Please explain ___<br>★ **Key Finding: Percentage sites reporting a smooth or very smooth introduction**
GEN	29. Many other countries will be introducing the HPV vaccine and other new vaccines soon. What have you learned from this experience and what advice do you have for other health facilities to ensure a smooth introduction?	

12. OBSERVATIONS AT VACCINATION SESSION	
30. How well is the site organized?	☐At least 2 trained health workers on the site?<br>☐At least one teacher available?<br>☐Is there a waiting area?<br>☐Is furniture organized?<br>☐Screening area available?<br>☐Registration area?
31. Are vaccines stored/handled properly during the session, e.g. clean, organized, vaccine vials outside carrier are in foam pad?	☐ Yes ☐ No ☐ Don't know<br>(N = unsafe practice)
32. Are appropriate administration techniques observed (e.g. HPV intramuscular injection in the deltoid region of the upper arm)?	☐ Yes ☐ No ☐ Don't know<br>(N = unsafe practice)
33. Is an emergency box present?	☐ Yes ☐ No
34. Did health worker observe the girl afterwards for 15 minutes after vaccination for any adverse reactions?	☐ Yes ☐ No<br>(N = unsafe practice)

35. Are AD syringes used?	☐ Yes ☐ No<br>(N = unsafe practice)
36. Are needles recapped (look in safety box for capped needles)?	☐ Yes ☐ No<br>(Y = unsafe practice)
37. Are AD syringes disposed of in a safety box?	☐ Yes ☐ No<br>(N = unsafe practice)
38. Summary: How many unsafe practices, based on questions above, were observed?	Number of unsafe practices________________________<br>★ **Key Finding: Percentage of sites with two or more unsafe practices observed**
**13. HEALTH COMMUNICATION**	
39. Are any posters or other literature about the HPV vaccine noted in the school/ health facility/outreach site?	☐ Yes ☐ No
40. What messages did the health worker provide? Give any health messages related to cervical cancer or its risks?	☐ Health messages related to cervical cancer or its risks?<br>☐ Health messages related to other health interventions?<br>☐ Health messages on other health issues?<br>☐ Other?
**14. WASTE DISPOSAL**	
41. How are used AD syringes being disposed of?<br><br>(If not observed, ask how AD syringes are disposed).	☐ safety box<br>☐ Open bucket<br>☐ Other containers, specify<br>☐ Other safety related observations<br>________________________________
42. Were discarded needles and syringes observed on the ground outside the facility?	☐ Yes ☐ No
43. How and where is waste disposed of?	
