# Supplementary material for: Nationwide introduction of HPV vaccine in Zimbabwe 2018–2019: Experiences with multiple cohort vaccination delivery
Source: PLOS Glob Public Health. 2022 Apr 6;2(4):e0000101. doi: 10.1371/journal.pgph.0000101 (PMC10021852; doi:10.1371/journal.pgph.0000101)
Supplement: Attachment — Submitted filename: ZimbabweHPV part 1 PLoS_response to reviewers2.docx [file pgph.0000101.s003.docx]

| **Reviewer Comments** | **Author Response** |
| --- | --- |
| *Editor Comments* | |
| 1. Please include a copy of the interview guide used in the study, in both the original language and English, as Supporting Information, or include a citation if it has been published previously. | The semi-structured interview guide used in the study has been included as supplementary material. This includes national-level interview questions for key informants as well as a questionnaire for health workers administered during a vaccination session as part of the WHO Post Vaccine Introduction Evaluation (PIE). The standardized WHO guidelines for this tool is cited in the methods (line 120) by reference 6: New Vaccine Post-Introduction Evaluation (PIE) Tool [Internet]. World Health Organization; 2010 [cited 2020 Jul 22]. Available from: https://apps.who.int/iris/bitstream/handle/10665/70436/WHO_IVB_10.03_eng.pdf. |
| 2. We note that participants provided oral consent. Please state in the Methods:   - Why written consent could not be obtained - How oral consent was documented | Oral consent was obtained prior to key informant interviews and vaccination session observations in line with standard local procedures and post-introduction evaluation guidelines and as outlined in the CDC ethical review board’s non-research determination and the Medical Research Council of Zimbabwe waiver. Oral informed consent was obtained by project staff prior to any key informant interviews and is documented on the interview guides. This information has been clarified in the methods in line 128. |
| 3. We noticed that you used “data not shown”/"unpublished data" in the manuscript. We do not allow these references, as the PLOS data access policy requires that all data be either published with the manuscript or made available in a publicly accessible database. Please either remove these references, or amend the supplementary material to include the referenced data. | Reference 5 (unpublished data) has been deleted. |
| 4. We do not publish any copyright or trademark symbols that usually accompany proprietary names, eg (R), (C), or TM  (e.g. next to drug or reagent names). Therefore please remove all instances of trademark/copyright symbols throughout the text, including CervarixTM on table 1. | The TM superscript has been removed from Cervarix in Table 1 |
| 5. In the online submission form, you indicated that "Excerpts of transcripts relevant to the study available upon request". All PLOS journals now require all data underlying the findings described in their manuscript to be freely available to other researchers, either 1. In a public repository, 2. Within the manuscript itself, or 3. Uploaded as supplementary information. This policy applies to all data except where public deposition would breach compliance with the protocol approved by your research ethics board. If your data cannot be made publicly available for ethical or legal reasons (e.g., public availability would compromise patient privacy), please explain your reasons by return email and your exemption request will be escalated to the editor for approval. Your exemption request will be handled independently and will not hold up the peer review process, but will need to be resolved should your manuscript be accepted for publication. One of the Editorial team will then be in touch if there are any issues. | Underlying data from health worker interview and vaccination session observation checklist have been deidentified and uploaded as supplementary information.  Transcripts from key informant interviews may be requested from Dr. Terri Hyde ([thyde@cdc.gov](mailto:thyde@cdc.gov))  Underlying data from key informant interviews were not provided in full in line with CDC and country ethical review guidelines but transcripts may be made available at request of the non-author contact listed above. |
| 6. Please amend your detailed Financial Disclosure statement. This is published with the article, therefore should be completed in full sentences and contain the exact wording you wish to be published.   i. State the initials, alongside each funding source, of each author to receive each grant. ii. State what role the funders took in the study. If the funders had no role in your study, please state: “The funders had no role in study design, data collection and analysis, decision to publish, or preparation of the manuscript.”   If you did not receive any funding for this study, please simply state: “The authors received no specific funding for this work.” | The grant supporting the project was provided to CDC Foundation for project implementation. Authors JGC, AH and AL were supported by this grant.  The following statement has been added to the paper  This work was supported by Gavi, the Vaccine Alliance [“Evaluation of Human Papilloma Virus (HPV) Vaccine National Introduction in Low-and-Lower-Middle Income Countries” - Contract No. ME 9422 12 20] through a grant to CDC Foundation for project implementation. The funders had no role in study design, data collection and analysis, decision to publish, or preparation of the manuscript. |
| Please review your reference list to ensure that it is complete and correct. If you have cited papers that have been retracted, please include the rationale for doing so in the manuscript text, or remove these references and replace them with relevant current references. Any changes to the reference list should be mentioned in the rebuttal letter that accompanies your revised manuscript. If you need to cite a retracted article, indicate the article’s retracted status in the References list and also include a citation and full reference for the retraction notice. | The reference list has been checked with minor formatting edits made. |
| *Reviewer 1 Comments* | |
| Abstract: Results Line 48-49 should be either broken up or explained more succinctly. The age-based vs. Grade-based is it's own topic (strategy). Funding shortages and sustainability are future challenges to the current approach, as then discussed in the conclusions paragraph? | Thank you for this suggestion. The sentence has been edited to refer to the challenges reported during the current campaign (transitioning target populations and funding shortages) in line 49-50. Sustainability is already addressed in the following paragraph and was removed from this paragraph for clarity. |
| Introduction: Line 75: »high school enrollment« is confusing for those from places high school is the term used for secondary school. Rephrasing will help clarify the ages/grades targeted for vaccinations. | This sentence has been rephrased to “high primary school enrollment” for clarity (line 76). |
| Results: This may not be available, but would improve the manuscript: what percentage of eligible girls were successfully vaccinated and how many were missed? | We have included the official WUENIC estimates of 83% coverage and 751,367 administered doses in line 212-213. We did not include the percent of number of girls missed as this figure is not reported in the countries official estimates. |
| Discussion: Lines 291-294: What are strategies that are less resource intensive that could be considered as an alternative to the school-based strategies? | To address this comment, the following sentence has been added to the discussion (line 291-292):  Alternative strategies to school-based platforms include health-facility vaccination and community outreach.  The concept of integration with other vaccination or school health activities is mentioned in the following paragraph, another known strategy to reducing the high costs associated with a stand-alone school-based delivery platform. |
| Line 304: While Zimbabwe is unique, why can’t some of these concepts be generalized amd used elsewhere for vaccine implementation strategy? | It is possible that some general concepts from this experience may be used for other countries planning HPV vaccine introduction in the future. This has been clarified in line 311-312. |
| *Reviewer 2 Comments* | |
| I have no required changes, just a pet thing about data "are" rather than data "is". | This error was corrected throughout the paper. |
